# Thoughts on Intermittent and Remittent Fever

**Published:** 1847-03

**Authors:** 


*Thoughts on Intermittent and Remittent fever*, by Isaac Branch, of Abbeville, S. C.

The following extract from an article by Dr. Branch, entitled as above, we select as evidence that views similar to those which we have more than once taked occasion to express, are not peculiar to ourselves.—*Ed. Buffalo Med. Jour.*

"When we are called to a case of nervous intermittent fever, as a matter of course we investigate the symptoms. If there be no derangement of the bowels—if we are informed that the last stool was a natural one, as to *color* and *consistency*, we are utterly at a loss to perceive any necessity for *producing*, or "getting up" a derangement by a resort to cathartics. As a matter of course then, we do not use them, and for the reason we reject emetics. The patient however, has a dry hot skin, a frequent pulse, a severe pain in the head, back, and lower joints, general restlessness, &c., and if the case be "congestive" (a word which we dislike, but which we are forced to use,) the extremities are cold, pulse not only frequent but extremely weak, laborious respiration, and sometimes a cold clammy sweat,—we say when we are called to a case of the first kind referred to, we are in the habit of meeting the symptoms promptly with free doses of camph. Dov. powder, and for this purpose we give from 2 to 15 or 20 grains, depending on the age and susceptibility of the patient, and the severity of the attack. We repeat this every hour until we produce free perspiration, when we commence with free doses of quinine, keeping up the determination to the surface with enough of the camph. Dov. for that purpose. In a day or two, if the bowels become constipated, we conjoin laxative doses of aloes and rhubarb, and sometimes a little calomel in the form of pill, never suspending the main treatment if the case be a bad one, if it be mild however, we sometimes do suspend the other treatment until after the operation. If the case be 'congestive,' we commence our treatment with free doses of quinine and the camphorated Dover's powder, say from 2 to 6 grs. of the former, and from 2 to 20 of the latter, giving at the same time alcohol in some form, capsicum either in substance or the oil, the oil of black pepper, or some other acrid, conjoining rubefacients to the surface. It will be perceived that we have not forgotten our text—we have only given the outline of our treatment.

"In relation to intermittent, we will simply remark that we have no fears of increasing fever by the use of quinine, and we do not believe there is one case in twenty that requires any preparatory treatment whatever. We will remark farther, that we believe this form of fever may be almost as effectually *prevented*, as it can be *cured*; when the incipient symptoms begin to "hang about" the system we generally "drive them away" with from 6 to 10 grains of quinine and a teaspoonful of capsicum at a single dose.

"It might be *almost* inferred, that we have sealed up our calomel jar, hermetically. This is a mistake. We use calomel, however, much more freely in other diseases, than in fever. We also use it in fever, whenever we conceive it to be necessary, we do not prescribe it, however, *as a mere matter of course because a patient has fever*. We prescribe it to meet some symptom, such as derangement of the bowels, &c., but if there be no symptom calling for its use, we confess we prefer letting it remain in the jar, where at least it can do no harm. We believe there are *pounds* of it used where *ounces* would produce less harm, and where *scruples* would answer a much better purpose, and upon this point we hope we shall not be considered egotistical when we affirm that we have treated in our opinion largely over one hundred cases of intermittent and remittent fever, (perhaps two hundred) within the last three or four weeks, and have not lost a case. We are confident, we have not prescribed 100 grains of calomel in the treatment of the whole of them."—*Southern Jour. of Med. and Pharmacy.*

